# Research on the cross-campus unsafe cycling behavior of university students based on cycling habits and risk perception

**DOI:** 10.3389/fpubh.2026.1788578

**Published:** 2026-04-28

**Authors:** Yingjie Wu, Tingting Hu, Zijun Liang

**Affiliations:** 1 Shanghai Communications Polytechnic, Shanghai, China; 2 School of Urban Construction and Transportation, Hefei University, Hefei, China

**Keywords:** cross-campus, structural equation modeling, theory of planned behavior, university students, unsafe cycling behavior

## Abstract

Cross-campus cycling among university students is increasingly common, yet unsafe cycling behaviors pose significant safety risks. Existing studies have rarely examined the combined roles of cycling habits and risk perception within a unified theoretical framework. This study aims to investigate the influencing mechanisms of unsafe cycling behavior among cross-campus university students based on an extended social-psychological model. An improved Theory of Planned Behavior (TPB) model incorporating cycling habits and risk perception was developed. A total of 492 valid questionnaires were collected from university students engaged in cross-campus travel. Structural Equation Modeling (SEM) was employed to test the proposed model and examine the relationships among variables. The results indicate that the improved TPB model shows significant associations with unsafe cycling behavior. Both cycling habits and risk perception exert direct and indirect effects within the model framework. Compared with the traditional TPB model, the improved model demonstrates a better fit and explains an additional 11% of the variance in behavior. These findings support the effectiveness and rationality of extending the TPB model to better understand unsafe cycling behavior. However, due to the cross-sectional design and reliance on self-reported data, causal relationships cannot be firmly established. The study provides practical insights for developing targeted interventions to improve cycling safety among university students.

## 1 Introduction

With the continuous improvement of China’s social-economic level, the number of non-motorized vehicles, especially electric bicycles, has been rapidly increasing. Due to their economic and convenient characteristics, electric bicycles have become one of the main tools for residents to short-distance travel. However, the road traffic safety issues associated with them have also become increasingly concerning. According to data from the Ministry of Public Security, from 2018 to 2022, there were 7,642 deaths and 66,861 injuries nationwide due to electric bicycle traffic accidents on urban roads, with direct economic losses reaching 120 million yuan (1). As the controllers of electric bicycles, unsafe cycling behaviors of riders are one of the main causes of road traffic accidents (2). Unsafe cycling behavior refers to the behavior that the cyclist subjectively considers himself or herself to be in a safe state, and then violates the traffic rules or has safety hazards, such as speeding (2), illegal lane occupation (3), running red lights (3), using a mobile phone during cycling (4), driving on the wrong side of the road (4), and not wearing a helmet (5). Analyzing unsafe cycling behavior is crucial for reducing traffic accidents and improving traffic safety.

Currently, some scholars have studied unsafe cycling behavior by constructing psychological models. For example, Yang et al. (6) integrated the Theory of Reasoned Action (TRA), Overconfidence Theory (OT), and Deterrence Theory (DT) to analyze the influencing factors of students’ mobile phone use while cycling electric bicycles. Tang et al. (7) adopted a modified Reinforcement Sensitivity Theory (RST) to investigate the factors influencing risky cycling behaviors of electric bicycle users from the perspective of traffic management’s reward and punishment mechanisms. Based on data collected through questionnaires, Wang et al. (8) used SEM to explore the influencing factors of risky cycling behavior. Although these studies employed various psychological models and explored several factors influencing unsafe cycling behavior, they did not consider the external constraints and individual capabilities that riders face when performing behaviors, which may result in insufficient explanatory power for unsafe cycling behavior.

The Theory of Planned Behavior (TPB) (9), proposed by social psychologist Ajzen in 1985, is an extension of the Theory of Reasoned Action (TRA). It introduces the variable of perceived behavioral control, which takes into account the external constraints and individual capabilities that individuals face when performing behaviors, thus improving the accuracy of behavioral predictions. The core idea of TPB is that attitude to behavior, subjective norm, and perceived behavioral control jointly influence behavioral intention, which in turn directly affects behavior. For example, Barton et al. (10) used the TPB to study pedestrian distraction while crossing the street; Wang et al. (11) applied TPB to investigate illegal lane-changing behavior of motorists at urban intersections; and Yang et al. (12) employed TPB to examine riders’ behavioral intentions to run red lights. With the continuous development of TPB, researchers have found that, in certain specific contexts, introducing new factors or integrating multiple models can enhance the explanatory power of the model. Therefore, the improved TPB model has been widely applied in the study of unsafe cycling behavior. For example, Zhang et al. (13) developed the improved TPB model by incorporating cycling habits, and the results showed that riders’ behavioral experience was significantly positively correlated with behavioral intention. Xie and Yang (2) introduced external perception into the TPB model and found that external perception had a significant positive relationship with behavioral intention and could directly influence behavior. Tang et al. (5) integrated the TPB and the Prototype Willingness Model (PWM), effectively explaining riders’ red-light running behavior. Wang et al. (14) combined the TPB, Protection Motivation Theory (PMT), and Psychological Reactance Theory (PRT), providing a better explanation for riders’ intentions to wear helmets.

With the expansion of higher education institutions, electric bicycles have become a primary mode of commuting on campus, and their riding safety has attracted increasing attention. However, existing studies have mainly focused on within-campus contexts. For example, Deng and Liu (15) examined environmental regulatory attitudes, while Xu et al. (16) investigated factors such as cycling anger. Nevertheless, insufficient attention has been paid to unsafe riding behavior across campuses. Under the multi-campus operational model, students frequently travel between different campuses separated by urban roads, and the riding context extends from internal campus roads to public roadways. Unsafe riding habits formed on campus may transfer to off-campus environments, where traffic conditions are more complex and safety risks are consequently higher. Therefore, students’ daily riding habits and their perception of potential risks on off-campus roads may have important influences on their cross-campus riding behavior.

In summary, although TPB has made progress in analyzing unsafe cycling behavior on urban roads, there is still a relative lack of research targeting university students—particularly their cross-campus cycling behavior—there are relatively few studies that examine the associated factors of unsafe cycling behaviors by integrating cycling habits, risk perception, and the TPB. Therefore, this study focuses on the unsafe cross-campus cycling behavior of university students and carries out the following two innovative contributions:

1. Going beyond the traditional TPB framework, this study incorporates both cycling habits and risk perception into an improved TPB model, aiming to analyze university students’ cross-campus unsafe cycling behavior from a social-psychological perspective.
2. Focusing on the cross-campus cycling context, the study develops a questionnaire tailored to university students’ cross-campus cycling behavior based on the improved TPB model. Using Shanghai Communications Polytechnic as a case study, data were collected and structural equation modeling (SEM) was used to quantitatively analyze the association pathways between various factors and unsafe cycling behavior, thereby providing decision-making support for the development of intervention strategies.

The remainder of this paper is organized as follows: Section 2 presents the research theory and model; Section 3 outlines the research methodology; Section 4 provides an analysis of the data; Section 5 discusses the reasons behind the results; and finally, Section 6 concludes the study.

## 2 Theoretical background and research model

### 2.1 The theory of planned behavior

The TPB model holds significant theoretical value in explaining and predicting unsafe cycling behavior. Numerous studies have shown that incorporating additional variables into the TPB framework can lead to a more comprehensive understanding and more accurate prediction of unsafe cycling behavior. For example, Deng and Liu (15) introduced the variable of environmental control attitude into the TPB model, Xie and Yang (2) added external perception to TPB, and Song and Yang (17) incorporated risk preference into TPB. These studies have proven that introducing variables into the TPB model can provide a more comprehensive understanding and prediction of unsafe cycling behavior. Therefore, we choose TPB as the theoretical framework to study the influence of attitude to behavior, subjective norm, perceived behavioral control, and behavioral intention on unsafe cycling behavior.

In this model, attitude to behavior (AB) refers to the rider’s perception of unsafe cycling behavior, including positive or negative evaluations. Subjective norm (SN) refers to the social pressure or expectations perceived by the rider when engaging in unsafe cycling behavior, including how their family, friends, or social groups view such behavior. Perceived behavioral control (PBC) refers to the rider’s perception of their ability to control unsafe cycling behavior. Behavioral intention (BI) refers to the rider’s willingness to perform unsafe cycling behavior. Behavior (B) refers to whether the rider actually engages in unsafe cycling behavior.

### 2.2 Cycling habits

Previous studies have shown that poor cycling habits are significantly associated with behavioral intention (BI) and behavior (B) (5, 13, 18) Earlier research has also explored the interactions between CH and the components of the TPB model, including AB, SN, and PBC. For example, in an improved TPB model, reference (5) while also considering the interrelationships among past behavior and attitudes, subjective norms, and perceived behavioral control. The study found that past behavior and AB were significantly positively correlated with PBC, but significantly negatively correlated with SN.

### 2.3 Risk perception

Previous studies have confirmed that RP is significantly associated with behavioral intention (BI) and behavior (B) (7, 18–20), and it is considered one of the critical factors influencing unsafe cycling behavior. Previous research has also considering the interrelationships between RP and AB, SN, and PBC. For example, in an improved TPB model, reference (18) simultaneously examined the mutual influences among RP, AB, SN, and PBC, and concluded that RP was negatively correlated with AB, showed no significant correlation with SN, and was negatively correlated with PBC.

### 2.4 The proposed research model

**Figure 1:**
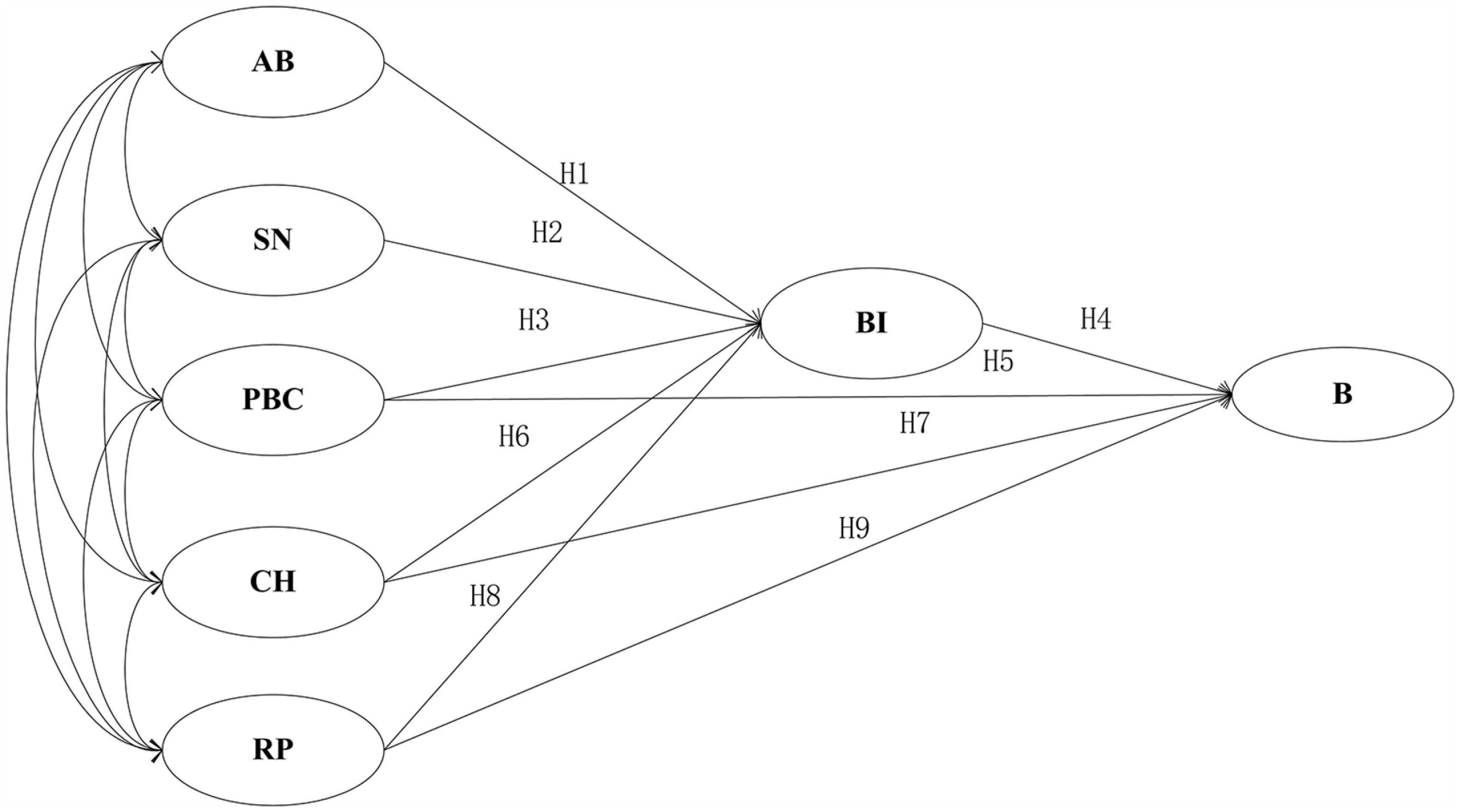
The improved TPB model.

Based on the traditional TPB model, CH and RP were introduced to construct an improved TPB model for studying the cross-campus unsafe cycling behavior of university students, as shown in Figure 1. The improved TPB model builds upon the traditional TPB framework by incorporating both the direct and indirect relationships of CH and RP with B, in order to enhance the model’s explanatory power for the cross-campus unsafe cycling behavior of university students, while also considering the interrelationships among CH, RP, AB, SN, and PBC (5, 18), in order to more comprehensively analyze the cross relationships between these factors. The model includes a total of nine hypothesized paths, which are explanation in Table 1.

**Table 1:** Hypothesis explanation of model path.

Hypothesis path	Hypothesis explanation
H1	AB was a significant positive associated with BI.
H2	SN was a significant positive associated with BI.
H3	PBC was a significant positive associated with BI.
H4	BI was a significant positive associated with B.
H5	PBC was a significant positive associated with B.
H6	CH was a significant positive associated with BI.
H7	CH was a significant positive associated with B.
H8	RP was a significant negative associated with BI.
H9	RP was a significant negative associated with B.

## 3 Method

### 3.1 Procedure

The experimental scheme was reviewed and approved by the Anhui Province Transportation Big Data Analysis and Application Engineering Laboratory on July 1, 2024. Expert committee of the laboratory concluded that the survey questionnaire would not cause any mental harm to the participants, nor would it have any negative impact. We have obtained informed consent from all subjects, and ensured that all methods were carried out in accordance with relevant guidelines and regulations. The research design was deemed scientifically rigorous, ensuring the terminology accurately reflects our survey-based methodology rather than an experiment. At the beginning of the survey, we introduced the purpose of the research and emphasized that all participants were volunteering, and that their responses would remain anonymous to ensure the efficiency of data collection and the privacy protection of the respondents.

**Figure 2:**
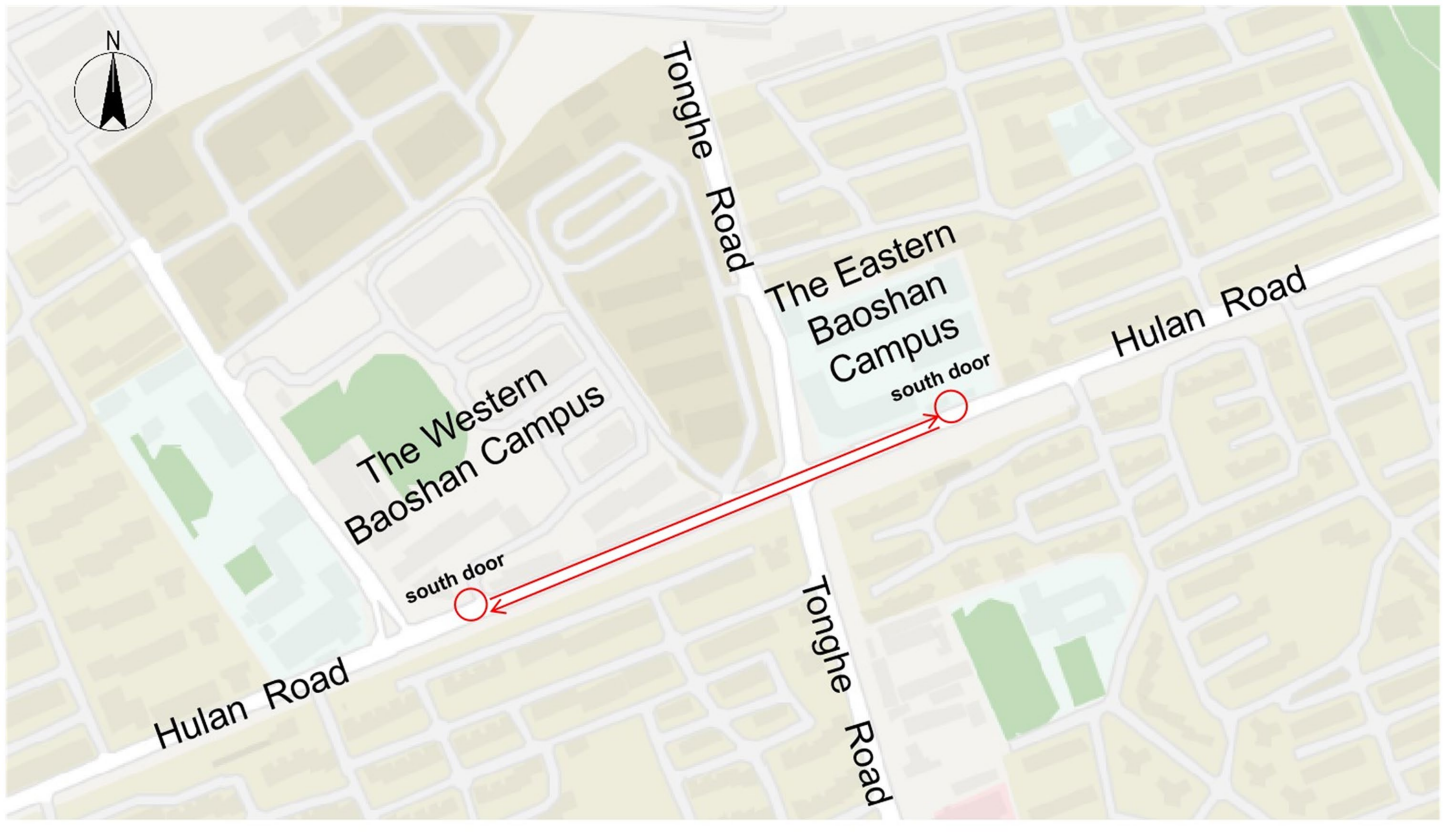
The Shanghai communications polytechnic.

The survey was conducted from July 9th to 19th, 2024. The survey site was Shanghai Communications Polytechnic, and the survey subjects were the college students on campus. As shown in Figure 2, Shanghai Communications Polytechnic is divided into two campuses by Tonghe Road, namely the Western Baoshan Campus and the Eastern Baoshan Campus. The two campuses are 800 meters apart. College students often use electric bicycles as a means of transportation, commuting between the two campuses via Hulan Road for classes, experimental activities, and other purposes. To efficiently collect sample data, we designed and distributed the questionnaire using the online survey platform “Wenjuanxing.” Participants could take part in the survey by simply clicking the provided link. The survey links are mainly distributed through the following channels: the class We Chat group of students majoring in transportation, the student community group of cycling enthusiasts, and the campus community forum. To ensure sample diversity, we set quotas for gender (about 50% male/female) and grade (freshman to senior and above), and asked each group coordinator to forward links to students who meet these quotas.

### 3.2 Participants

The total number of students at Shanghai Communications Polytechnic is 5,760. In this survey, 510 samples were collected, resulting in a sampling rate of 8.85%, after screening out responses with extremely short completion times, identical answers, incomplete content, or obvious issues, 492 valid questionnaires were retained, yielding a valid response rate of 96.5%. According to research, the required sample size for a questionnaire survey should be at least five times the number of items (21). With 26 items in the questionnaire, the minimum required sample size is 130. The final collection of 492 valid questionnaires meets this requirement.

By fully considering demographic characteristics such as gender, in-school period, and cycling experience, a stratified random sampling method was employed to ensure that the sample matched the overall student population in key dimensions such as gender ratio and duration of enrollment. The campus distribution and student travel patterns of Shanghai Communications Polytechnic are representative. Preliminary investigations revealed that many other universities share similar situations; therefore, the results of this survey can serve as a reference for institutions with comparable campus layouts and student commuting patterns, demonstrating a certain representativeness. The sample in this study was drawn from a single university, and the results may be influenced by specific campus layout, traffic environment, and local cycling culture.

### 3.3 Measures

The survey questionnaire consists of two parts. The first part primarily investigates personal attribute variables, including gender, in-school period, cycling experience, cycling frequency, purpose of cycling, type of electric bicycle, understanding of traffic regulations, electric bicycle registration and traffic accident experience. The second part mainly investigates the variables in the improved TPB model, including AB, SN, PBC, CH, RP, BI, and B. As shown in Table 2, each factor is designed with 3–6 items, for a total of 26 items. Each item is evaluated using a Likert 5-point scale, where the range of responses is from 1 (strongly disagree) to 5 (strongly agree).

**Table 2:** Scale content.

Variable	Item	Content	Reference
AB	AB1	I believe that occasional dangerous cycling can bring me convenience while ensuring my own safety.	Tang et al. (5)
	AB2	I believe that whether to follow traffic rules while cycling can be determined based on the situation.	
	AB3	In special circumstances, such as being in a hurry to class, I believe that occasional dangerous cycling behavior can save me time.	
SN	SN1	My family approves of my occasional dangerous cycling behavior while ensuring my own safety.	Tang et al. (5)
	SN2	My teachers approve of my occasional dangerous cycling behavior while ensuring my own safety.	
	SN3	My classmates approve of my occasional dangerous cycling behavior while ensuring my own safety.	
PBC	PBC1	I believe I can keep a close eye on road conditions.	Deng and Liu (15)
	PBC2	I believe I can react in time and control the brakes in emergency situations.	
	PBC3	When the green light at an intersection is about to end and there is no traffic, I believe I can pass through the intersection quickly and safely.	
CH	CH1	Sometimes to save time, I am accustomed to riding very fast in the open campus.	Wang et al. (14)
	CH2	When there is no clear regulation on wearing helmets in the campus, I am accustomed to not wearing a helmet.	
	CH3	When cycling in the campus, I am accustomed to weaving between vehicles and pedestrians.	
	CH4	If my phone rings or vibrates during cycling, I am accustomed to checking my phone while riding.	
	CH5	I am accustomed to turning without observing the rear situation in the campus.	
RP	RP1	With no vehicles passing through the intersection and ensuring my own safety, the risk of collision accidents from running red lights is relatively high.	Tang et al. (7)
	RP2	Without exceeding the speed limit and ensuring my own safety, the risk of serious injury from not wearing a helmet is relatively high.	
	RP3	When the non-motorized lane is in poor condition and ensuring my own safety, the risk of serious collision accidents from cycling in the motorized lane is relatively high.	
BI	BI1	When there are no vehicles passing through the intersection and to save time while ensuring my own safety, I would choose to run the red light.	Wang et al. (8)
	BI2	With the premise of ensuring my own safety, I would choose not to wear a helmet.	
	BI3	When the non-motorized lane is in poor condition and there are few vehicles in the motorized lane, I would choose to cycle in the motorized lane.	
B	B1	When in a hurry to class in phase one, two, or three of the campus, I would accelerate through the intersection at the last of the green light.	Deng and Liu (15)
	B2	When cycling, I do not wear a helmet if there are no traffic police.	
	B3	When the non-motorized lane is in poor condition, I cycle in the motorized lane.	
	B4	To conveniently enter the campus, I cycle against the traffic in the non-motorized lane.	
	B5	I use my phone while cycling (such as answering calls, checking messages, etc.).	
	B6	I turn without observing the rear situation while cycling.	

Some items (e.g., AB1, AB3, and BI1) include expressions such as “under the premise of ensuring one’s own safety” or “in order to save time.” This design is based on findings from preliminary interviews, which revealed that students often use such reasons to justify unsafe cycling behavior. However, this wording may introduce certain limitations, such as justification bias and social desirability bias, thereby potentially affecting respondents’ true assessment of risk.

## 4 Results

### 4.1 Descriptive statistics

The descriptive statistics and difference analysis results of the personal attribute factors of college student cyclists are shown in Table 3.

**Table 3:** Descriptive statistics and difference analysis (*N* = 492).

Personal attribute factors	Categories	Number of people	Proportion	Mean (SD)	*F* (*t*)	*p*
Gender	Male	201	40.9%	2.2736 (1.00071)	1.098	0.091
	Female	291	59.1%	2.1775 (0.92101)		
In-school period	Less than 1 year	95	19.3%	2.1509 (0.94502)	0.310	0.818
	1-2 years	183	37.2%	2.2368 (0.99638)		
	2-3 years	120	24.4%	2.2639 (0.95354)		
	3-4 years	94	19.1%	2.1844 (0.89017)		
Cycling experience	Less than 1 year	60	12.2%	2.1222 (0.90034)	1.087	0.354
	1-2 years	75	15.2%	2.0689 (0.80029)		
	2-3 years	63	12.8%	2.2725 (0.95944)		
	Over 3 years	294	59.8%	2.2619 (0.99848)		
Cycling frequency	1-2 times	166	33.73%	1.9910 (0.88311)	10.412	<0.001
	3–5 times	140	28.45%	2.1821 (0.82402)		
	Almost every day	186	37.82%	2.444 (1.05533)		
Cycling purpose	Going to classes or self-study	279	56.71%	2.2431 (0.95079)	0.163	0.921
	Going to eat	35	7.11%	2.1857 (0.97917)		
	Shopping or picking up parcels	106	21.54%	2.1808 (0.98511)		
	Leisure and entertainment	72	14.64%	2.1829 (0.92782)		
Electric bicycle type	Purchased new bike	191	38.83%	2.2234 (1.00764)	3.560	<0.05
	Purchased used bike	167	33.94%	2.3433 (0.89971)		
	Shared electric bicycle	134	27.23%	2.0498 (0.92447)		
Understanding of traffic regulations	Not very familiar	44	8.94%	2.2159 (0.87794)	3.748	<0.05
	Somewhat familiar	220	44.71%	2.3424 (0.91532)		
	Fairly familiar	165	33.53%	2.1697 (0.96386)		
	Very familiar	63	12.82%	1.9021 (1.04968)		
Electric bicycle licensing	Yes	464	94.3%	2.2083 (0.94963)	0.801	0.582
	No	28	5.7%	2.3571 (1.04104)		
Traffic accident experience	Yes	36	7.3%	2.4954 (1.07459)	1.823	0.426
	No	456	92.7%	2.1948 (0.94223)		

Among the 492 surveyed university students, 201 were male (40.9%) and 291 were female (59.1%), the majority of students had been in school for 1-2 years (37.2%), had more than 3 years of cycling experience (59.8%), and cycled almost daily (37.82%). Most of the university student cyclists had purchased electric bicycles (72.77%), and their main purpose for cycling was to attend classes or study (56.71%). The vast majority of electric bicycles were registered (94.3%), but more than half of the university student cyclists did not have sufficient knowledge of traffic regulations (53.65%). Additionally, most university student cyclists had a good record in terms of traffic accidents, with 92.7% of participants reporting no traffic accidents.

An independent samples *t*-test was conducted with the cross-campus unsafe cycling behavior of university students as the dependent variable to examine the differences between the binary variables: gender, electric bicycle registration, and traffic accident experience. For the multi-category variables (in-school period, cycling experience, cycling frequency, cycling purpose, electric bicycle type, and understanding of traffic regulations), analysis of variance was performed.

The analysis results show significant differences in the unsafe cross-campus cycling behavior of university students across different cycling frequencies (*p* < 0.001). Students who cycle almost every day are more likely to engage in unsafe cross-campus cycling behavior. There are also significant differences cross-campus unsafe cycling behavior of university students based on the type of electric bicycle (*p* < 0.05), with those who own second-hand bikes being more prone to unsafe cycling behavior. The understanding of traffic regulations is also significantly associated with unsafe cycling behavior (*p* < 0.05), with students who have a general understanding of traffic regulations being more likely to engage in unsafe cycling behavior. Additionally, no significant differences were found in unsafe cycling behavior based on gender, in-school period, cycling experience, cycling purpose, electric bicycle registration, or traffic accident history (*p* > 0.05).

### 4.2 Reliability and validity analysis

Reliability is an important indicator for measuring the consistency and internal consistency of a scale, and Cronbach’s *a* coefficient is the main evaluation index for assessing reliability. The value of Cronbach’s *a* ranges from 0 to 1, with values closer to 1 indicating higher internal consistency and reliability of the scale, thus making the results more reliable. For subscales, a Cronbach’s *a* coefficient greater than 0.7 indicates high reliability, while a coefficient between 0.6 and 0.7 is acceptable. For the total scale, a Cronbach’s *a* coefficient greater than 0.8 indicates high reliability, and a coefficient between 0.7 and 0.8 is acceptable (22). In this study, reliability analysis of each subscale and the total scale was conducted using SPSS 27.0, and the results are shown in Table 4. The Cronbach’s *a* coefficients for the seven subscales ranged from 0.855 to 0.931, all greater than 0.7, indicating high internal consistency. The Cronbach’s *a* coefficient for the total scale was 0.872, greater than 0.8, indicating good reliability and high consistency of the scale.

**Table 4:** Reliability analysis.

Scale	Item	Cronbach’s *a* coefficient
AB	3	0.867
SN	3	0.890
PBC	3	0.931
CH	5	0.897
RP	3	0.894
BI	3	0.855
B	6	0.916
Total Scale	26	0.872

Validity is an indicator of the effectiveness and correctness of the questionnaire items, in a scale, typically including content validity and construct validity. The higher the validity of the scale, the more reliable the measurement results are. The content validity of a scale is mainly assessed by expert review. In this study, the survey questionnaire was not only based on previous research but also repeatedly modified and adjusted to reflect the actual situation of cross-campus cycling among university students. Therefore, it can be considered that the scale in this study has good content validity. Construct validity is generally tested using factor analysis. Factor analysis was performed on the data from 492 samples to examine whether the questionnaire items in the improved TPB model variables in the items were closely related. Before conducting the factor analysis, Bartlett’s test of sphericity and the Kaiser-Meyer-Olkin (KMO) test were used to check whether the scale was suitable for factor analysis. When the KMO value is higher than 0.8 and Bartlett’s test is significant (*p* < 0.05), factor analysis can be performed (23). The KMO value was 0.895, and Bartlett’s test was significant (*p* < 0.001), indicating that there are common factors among the questionnaire items correlation matrices, and factor analysis is appropriate.

Exploratory factor analysis (EFA) was performed by principal component analysis and variance maximum rotation method. Items with a factor loading of less than 0.50 and a commonness of less than 0.45 were eliminated, following the conventional criteria for scale development. The final solution to the retained items explained about 60% of the total variance, which was considered sufficient in behavioral research (24). The results of the analysis are shown in Table 5. Seven factors were extracted, with the communalities of the items being greater than 0.45, the factor loadings being greater than 0.5, and the cumulative variance contribution rate being 77.72%, which is greater than 60%. This indicates that the factor analysis was effective. Based on the specific measurement items under each factor, the seven factors were named as follows: AB, SN, PBC, CH, RP, BI, and B.

**Table 5:** Factor variables contribution rates and questionnaire items loading.

Variable	Contribution rate	Item (loading)	Item (loading)	Item (loading)	Item (loading)	Item (loading)	Item (loading)
AB	33.997%	AB1 (0.817)	AB2 (0.819)	AB3 (0.840)	—	—	—
SN	45.078%	SN1 (0.860)	SN2 (0.862)	SN3 (0.793)	—	—	—
PBC	54.241%	PBC1 (0.898)	PBC2 (0.867)	PBC3 (0.883)	—	—	—
CH	61.682%	CH1 (0.845)	CH2 (0.807)	CH3 (0.827)	CH4 (0.778)	CH5 (0.832)	—
RP	67.939%	RP1 (0.865)	RP2 (0.896)	RP3 (0.857)	—	—	—
BI	73.063%	BI1 (0.771)	BI2 (0.786)	BI3 (0.838)	—	—	—
B	77.722%	B1 (0.784)	B2 (0.735)	B3 (0.777)	B4 (0.831)	B5 (0.787)	B6 (0.821)

Since all variables in this study were collected through the same questionnaire at a single point in time, there may be a risk of common method bias (CMB). To assess this issue, Harman’s single-factor test was employed. All 26 measurement items were subjected to an unrotated exploratory factor analysis, with one factor forcibly extracted, and the variance explained by the first factor was examined. The results of the unrotated single-factor analysis show that the first factor accounts for 33.997% of the total variance, in Table 6, which is below the critical threshold of 40%. Therefore, the results of Harman’s single-factor test indicate that there is no serious common method bias in this study.

**Table 6:** Total variance explained by Harman’s single-factor test.

Items	Initial eigenvalues		
	Total	Percentage of variance	Cumulative %
1	8.839	33.997	33.997
2	2.881	11.081	45.078
3	2.382	9.163	54.241
4	1.935	7.441	61.682
5	1.627	6.257	67.939
6	1.332	5.124	73.063
7	1.211	4.659	77.722
8	0.576	2.215	79.937
9	0.489	1.880	81.817
10	0.422	1.623	83.440
11	0.411	1.582	85.023
12	0.371	1.426	86.448
13	0.352	1.355	87.803
14	0.345	1.327	89.130
15	0.342	1.317	90.447
16	0.303	1.166	91.614
17	0.291	1.118	92.731
18	0.276	1.060	93.792
19	0.262	1.007	94.799
20	0.246	0.946	95.745
21	0.222	0.856	96.601
22	0.206	0.790	97.392
23	0.193	0.741	98.132
24	0.182	0.701	98.834
25	0.169	0.649	99.482
26	0.135	0.518	100.000

### 4.3 Correlation analysis

This study used Pearson correlation coefficient (*r*) to test the correlation between the original variables in the TPB model and the newly added variables, providing a theoretical basis for subsequent SEM analysis. The results are shown in Table 7. CH has a significant positive correlation with AB, SN, PBC, BI, and B, with *r* of 0.333 (*p* < 0.01), 0.286 (*p* < 0.01), 0.195 (*p* < 0.01), 0.312 (*p* < 0.01), and 0.320 (*p* < 0.01), respectively. RP has a significant negative correlation with AB, SN, PBC, BI, and B, with *r* of −0.293 (*p* < 0.01), −0.320 (*p* < 0.01), −0.195 (*p* < 0.01), −0.220 (*p* < 0.01), −0.327 (*p* < 0.01), and −0.334 (*p* < 0.01), respectively. In conclusion, all the variables in the improved TPB model are significantly correlated, these results validate the appropriateness of the improved TPB model in this study, which considers the relationships among CH, RP, AB, SN, PBC, BI, and B. Therefore, it is reasonable to use SEM to analyze the association pathways of the cross-campus unsafe cycling behavior of university students.

**Table 7:** Correlation analysis.

Variable	Mean (SD)	AB	SN	PBC	CH	RP	BI	B
AB	2.1883 (1.09256)	1						
SN	2.0217 (0.99147)	0.491 (<0.01)	1					
PBC	2.6809 (1.39748)	0.229 (<0.01)	0.307 (<0.01)	1				
CH	2.0907 (0.97696)	0.333 (<0.01)	0.286 (<0.01)	0.195 (<0.01)	1			
RP	3.5623 (1.14108)	−0.293 (<0.01)	−0.320 (<0.01)	−0.195 (<0.01)	−0.220 (<0.01)	1		
BI	2.2757 (1.07559)	0.389 (<0.01)	0.431 (<0.01)	0.378 (<0.01)	0.312 (<0.01)	−0.327 (<0.01)	1	
B	2.2168 (0.95454)	0.343 (<0.01)	0.329 (<0.01)	0.470 (<0.01)	0.320 (<0.01)	−0.334 (<0.01)	0.484 (<0.01)	1

### 4.4 Structural equation modeling

This study used AMOS 26.0 to establish a SEM, which consists of two parts: the first part is the measurement model formed by 7 latent variables and their corresponding 26 observed variables; the second part is the structural model that describes the inter relationships among the seven latent variables. During the model evaluation phase, the model’s overall fit was assessed based on eight inter nationally recognized fit indices and standards (25). These fit indices are used to measure the degree of fit between the theoretical model and the actual statistical data model, thereby evaluating the model’s adaptability to the data. The fit indices evaluation results are shown in Table 8. The results indicate that all the fit indices of the improved TPB model meet the standard values, and the overall model fit is good. This suggests that the hypothesized model fits well with the sample data, making the model acceptable. Therefore, the fitted SEM can be further used to analyze the causal relationships among the latent variables.

**Table 8:** Model fit assessment.

Model fit indicator	CMIN/DF	RMSEA	SRMR	AGFI	GFI	NFI	CFI	IFI
Criterion	1~3	<0.08	<0.08	>0.9	>0.9	>0.9	>0.9	>0.9
Improved TPB model	1.849	0.042	0.0353	0.927	0.946	0.960	0.980	0.980
Traditional TPB model	1.951	0.044	0.0398	0.905	0.924	0.942	0.972	0.972

**Figure 3:**
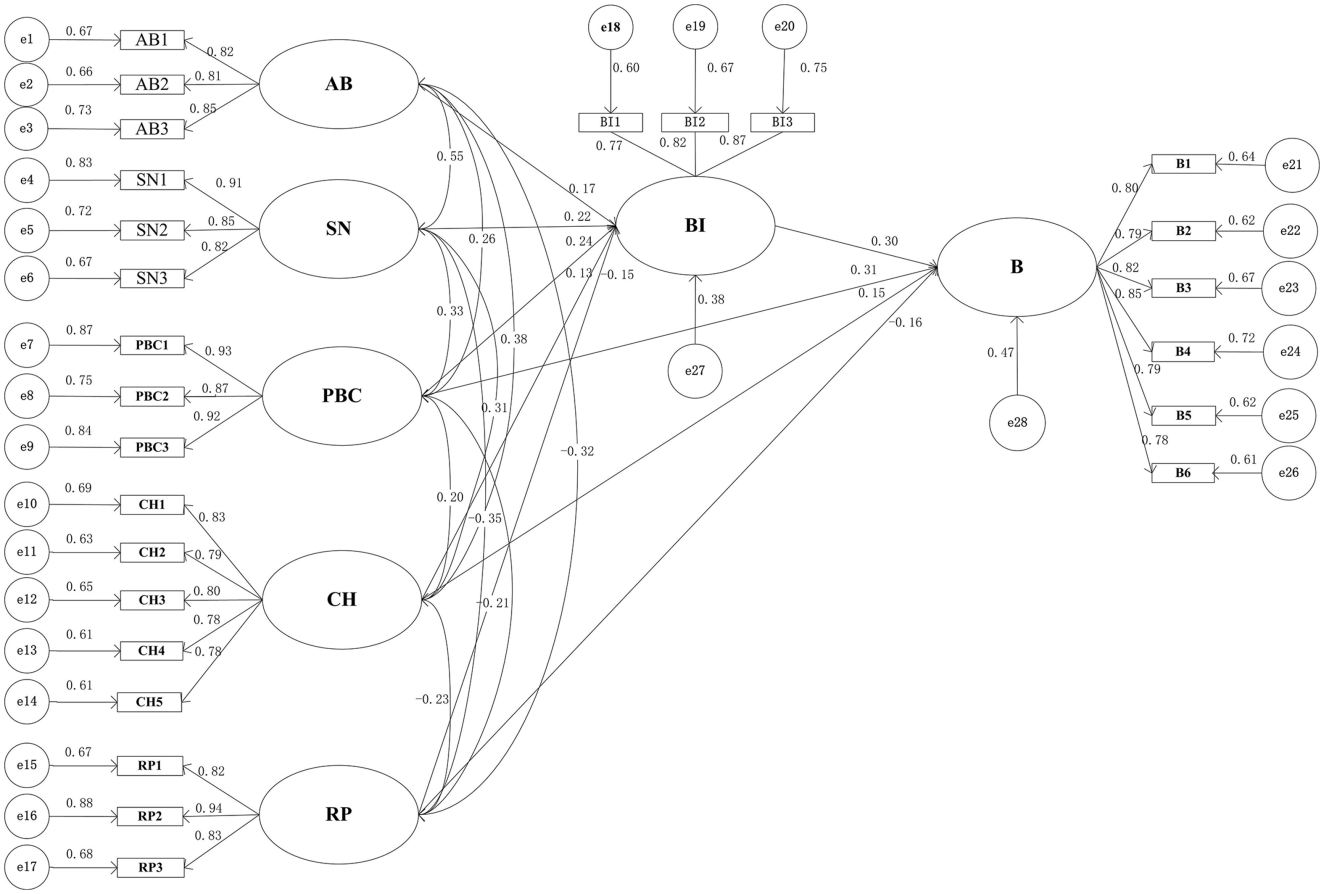
Path diagram of the SEM for the improved TPB model.

The maximum likelihood estimation method was used to estimate the path coefficients. The sign of the standardized path coefficient (*β*) was used to determine the direction of the relationship between variables, and the absolute value of *β* was used to reflect the relative strength of the association between variables. A larger absolute value of *β* indicates a stronger association between variables along the path. The standardized path coefficients and hypothesis testing results are shown in Table 9, and the structural equation model path diagram is shown in Figure 3. From Table 9 and Figure 3, it can be seen that all error variances (*e*) in the structural equation model are positive and significant (*t*-value >1.96, *p* < 0.01), with small standard errors (S.E.) for the parameter estimates. The standardized factor loadings range from 0.5 to 0.95, and there are no estimation violations, indicating that the model’s internal fit is ideal (26). Further hypothesis testing shows that AB (*β* = 0.148, *p* < 0.001), SN (*β* = 0.195, *p* < 0.001), PBC (*β* = 0.152, *p* < 0.001), CH (*β* = 0.127, *p* < 0.01), and RP (*β* = −0.133, *p* < 0.001) have statistically significant effects on BI, supporting hypotheses H1, H2, H3, H6, and H8. Additionally, BI (*β* = 0.306, *p* < 0.001), PBC (*β* = 0.203, *p* < 0.001), CH (*β* = 0.143, *p* < 0.01), and RP (*β* = −0.140, *p* < 0.001) have statistically significant effects on B, supporting hypotheses H4, H5, H7, and H9.

**Table 9:** Standardized path coefficients, hypothesis testing results, and *R*^2^.

Hypotheses	Path	Estimate	*p*	S.E.	C.R.	Results	*R* ^2^	
							BI	B
Improved TPB model							0.42	0.53
H1	AB → BI	0.148	<0.001	0.049	3.038	Supported		
H2	SN → BI	0.195	<0.001	0.051	3.823	Supported		
H3	PBC → BI	0.152	<0.001	0.030	5.155	Supported		
H4	BI → B	0.306	<0.001	0.052	5.894	Supported		
H5	PBC → B	0.203	<0.001	0.029	7.017	Supported		
H6	CH → BI	0.127	<0.01	0.047	2.699	Supported		
H7	CH → B	0.143	<0.001	0.043	3.342	Supported		
H8	RP → BI	−0.133	<0.001	0.041	−3.245	Supported		
H9	RP → B	−0.140	<0.001	0.038	−3.667	Supported		
Traditional TPB model							0.35	0.42
H1	AB → BI	0.206	<0.001	0.048	4.309	Supported		
H2	SN → BI	0.243	<0.001	0.052	4.697	Supported		
H3	PBC → BI	0.167	<0.001	0.030	5.540	Supported		
H4	BI → B	0.410	<0.001	0.051	8.114	Supported		
H5	PBC → B	0.216	<0.001	0.030	7.227	Supported		

The *R*^2^ value was further evaluated through the SEM *R*^2^ represents the proportion of variance in the dependent variable explained by the independent variables and is an important indicator of model fit. The range of *R*^2^ is between [0, 1], with values closer to 1 indicating a better model fit, and values closer to 0 indicating a poorer fit. Generally, an *R*^2^ greater than 0.45 indicates a good explanatory power of the independent variables for the dependent variable (27). The results in Table 9 show that the improved TPB model is significantly associated with the cross-campus unsafe cycling behavior of university students. The model explains 42% of the variance in BI and 53% of the variance in B. This indicates that after considering the direct and indirect effects of CH and RP on behavior, the explanatory power of the model for the cross-campus unsafe cycling behavior of university students is relatively good.

**Figure 4:**
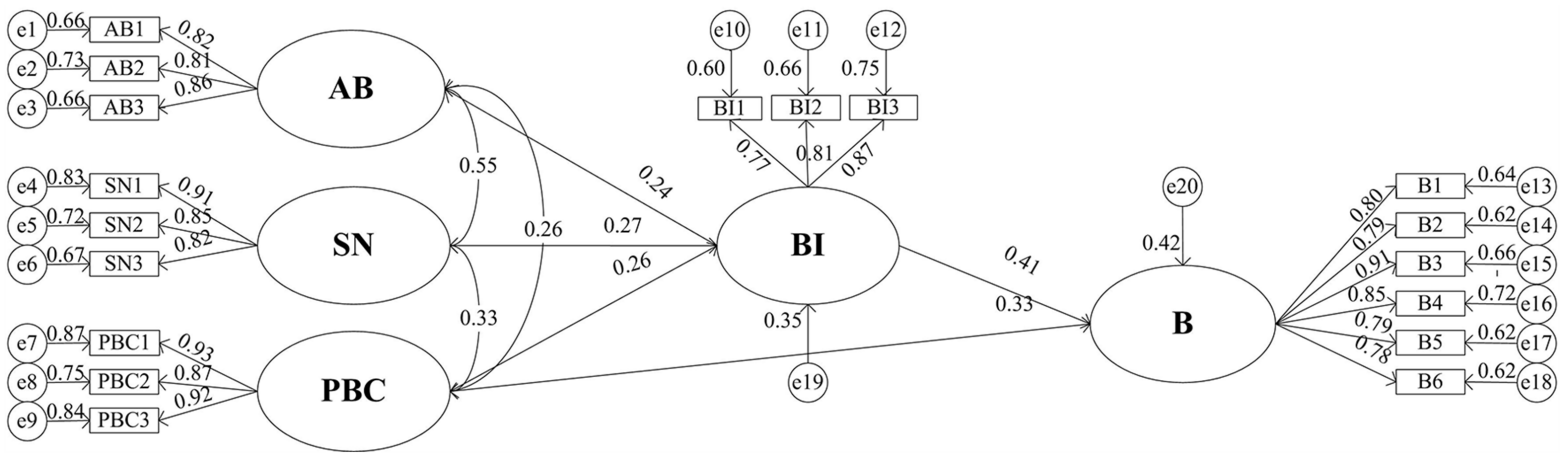
Path diagram of the SEM for the traditional TPB model.

In addition, a further analysis of the traditional TPB model was conducted. The model fit indices are shown in Table 8, and the standardized path coefficients, hypothesis testing results, and *R*^2^ values are presented in Table 9. The SEM path diagram is shown in Figure 4. The fit indices of the traditional TPB model met the required criteria, and the conclusions regarding the associations between various factors and behavior were generally consistent. The explanatory power for BI variance was 35%, and for B variance, it was 42%. When comparing the improved TPB model with the traditional TPB model, the results indicate that the fit indices of the improved model, including CMIN/DF, RMSEA, and SRMR, improved by 5.2, 4.5, and 11.3%, respectively. The explanatory power for BI variance increased by 7%, and the explanatory power for B variance increased by 11%. This indicates that the model in this study considers the direct and indirect relationships between CH and RP and B. The improved model fits the sample data of cross-campus cycling behavior of university students better and enhances the model’s explanatory power for the cross-campus unsafe cycling behavior of university students.

The improved TPB model increases the explanatory variance *R*^2^ of unsafe cycling behavior from 42% to 53% by adding new variables CH and RP on the basis of the traditional TPB model, with an increase of 11%. This significant improvement indicates that adding more predictors to the same questionnaire may improve *R*^2^ to a certain extent. Cycling habits and risk perception capture a unique psychological mechanism that is not covered by traditional TPB, that is, many unsafe behaviors when people cycling are not the result of rational thinking, but are pushed by some subconscious psychological factors, such as weak risk perception (6, 7). However, it must be admitted that 47% of the variance is still not explained. This shows that other factors (5, 7, 10) road conditions, road infrastructure or emotional state, etc., also play a role in explaining the variance of behavior, which is worthy of further study in the future.

## 5 Discussion

### 5.1 Associated with factors of the cross-campus unsafe cycling behavior intention

Path coefficient analysis of the SEM indicates that in the cross-campus context, AB (*β* = 0.148, *p* < 0.001), SN (*β* = 0.195, *p* < 0.001), and PBC (*β* = 0.152, *p* < 0.001) are all positively correlated with BI. This finding is consistent with the conclusions of previous studies by Tang et al. (5), Yang et al. (12), and Deng and Liu (15). For example, Tang et al. (5) found that AB and PBC are positively associated with behavioral intention; Yang et al. (12) reported that AB and PBC are positively correlated with cyclists’ intentions to run red lights; Deng and Liu (15) indicated that AB, SN, and PBC are significantly positively associated with behavioral intention; Xia et al. (28) found that AB is also significantly positively associated with intentions related to mobile phone use while cycling and cycling against traffic. Therefore, safety improvement measures for cross-campus cycling students should focus on improving attitudes toward unsafe cycling behavior, encouraging positive interventions from family, teachers, and peers, reducing PBC over unsafe cycling, and inhibiting the intention to engage in unsafe cycling, thereby reducing the frequency of unsafe cross-campus cycling behavior of university students. An improved TPB model for studying the cross-campus unsafe cycling behavior.

### 5.2 Associated with factors of the cross-campus unsafe cycling behavior

#### 5.2.1 Descriptive variable analysis

This study found significant differences in the cross-campus unsafe cycling behavior of university students based on cycling frequency (*p* < 0.001), type of electric bicycle (*p* < 0.05), and understanding of traffic regulations (*p* < 0.05). These findings are consistent with those of Deng and Liu (15), who found a significant difference in daily cycling duration and the cross-campus unsafe cycling behavior of university students. This is consistent with the findings of Xia et al. (28), who reported that cycling frequency is significantly associated with mobile phone use and cycling against traffic. However, the finding that the type of electric bicycle was not significantly correlated with unsafe cycling behavior in campus environments contradicts Deng and Liu’s conclusion (15). One possible explanation is that there are differences in electric bicycle usage habits, regional culture and other aspects of the research samples, which may lead to inconsistencies in the association between electric vehicle types and unsafe cycling behaviors across different samples. Additionally, the study’s finding that cycling frequency was significantly correlated with unsafe cycling behavior is inconsistent with Tang et al.’s (7) conclusion, which found no significant correlation between cycling frequency and risky cycling behavior. This difference may be due to the fact that most of the respondents in this survey had high cycling frequency and were experienced riders, so the differences in cycling frequency among individuals were not significant, and therefore cycling frequency did not have a notable impact on risky cycling behavior.

There were no significant differences in the cross-campus unsafe cycling behavior of university students based on gender, in-school period, cycling experience, cycling purpose, electric bicycle registration, or traffic accident history (*p* > 0.05). This is consistent with the findings of Deng and Liu (15), who reported that personal attributes such as gender and electric bicycle registration are not significantly associated with the cross-campus unsafe cycling behavior of university students; it is inconsistent with their finding that students with prior traffic accident experience are more likely to engage in unsafe cycling behaviors; it is also inconsistent with the findings of Xia et al. (28), who reported that gender, age, and educational level are negatively associated with unsafe cycling behaviors; and with Xu et al. (29, 30), who found that gender, age, and educational level are associated with red-light running behavior. One possible explanation is that men usually have stronger risk-taking tendencies and competition awareness. That is, with the increase of age and education. One possible explanation is that students who have experienced traffic accidents may have developed some bad habits and failed to correct their behavior after the incident. Instead, their behavior might be reinforced by a sense of invincibility or luck, making them more prone to unsafe cycling behavior.

#### 5.2.2 PBC and BI

Within the TPB framework, PBC and BI are associated with unsafe cycling behaviors. Based on the SEM path coefficient analysis, PBC (*β* = 0.203, *p* < 0.001) and BI (*β* = 0.306, *p* < 0.001) are significantly positively associated with B. This is consistent with the findings of Tang et al. (5), who reported that behavioral intention is positively associated with cyclists’ red-light running behavior; Deng and Liu (15), who found that PBC and BI are also significantly positively associated with risky cycling behaviors among university students on campus; Xia et al. (28), who found that PBC is significantly positively associated with behaviors such as speeding and cycling in motor vehicle lanes or on sidewalks; and Xu et al. (29, 30), who found that PBC is positively associated with red-light running behavior. The stronger the students’ intention to engage in unsafe behavior and the higher their PBC, the more likely they are to exhibit unsafe cycling behavior. This highlights the strong explanatory and predictive power of BI and PBC regarding unsafe cycling behavior.

#### 5.2.3 CH and RP

CH show a significant positive correlation with behavioral intention (BI) (*β* = 0.127, *p* < 0.001), and also a significant positive correlation with behavior (B) (*β* = 0.143, *p* < 0.001). These findings are consistent with those reported by Zhang et al. (13) and Tang et al. (18). For example, Zhang et al. (13) found that cyclists’ habits are significantly positively correlated with BI, while Tang et al. (18) reported that past violation behaviors are positively correlated with the intention of unsafe cycling. However, neither study (13, 18) considered the indirect and direct relationships between CH and B. In addition, some studies present differing viewpoints, for example, Wang et al. (14) concluded that cyclists who have a habit of wearing helmets are less likely to engage in risky cycling behaviors. This divergence arises because Wang et al. (14) focused on proper cycling habits, whereas this study emphasizes improper cycling behaviors. College students with long-term habits of running red lights or speeding are more likely to develop unsafe cycling intentions and act upon them.

RP is significantly negatively correlated with BI (*β* = −0.133, *p* < 0.001), and also significantly negatively correlated with B (*β* = −0.140, *p* < 0.001). These results are similar to those of Tang et al. (7) and Tang et al. (18). For instance, Tang et al. (7) found that risk perception has both indirect and direct negative correlations with risky cycling behavior, while Tang et al. (18) reported a negative correlation between risk perception and the intention of unsafe cycling. Liang et al. (31) also found that risk perception is negatively correlated with dangerous driving behavior among truck drivers. However, existing studies have certain limitations; for example, studies (7, 18) did not consider the indirect and direct relationships between risk perception and behavior.

This study extends the TPB framework (i.e., AB, SN, and PBC) by incorporating CH and RP, with the aim of enhancing the theoretical applicability of TPB in explaining unsafe cycling behavior. Specifically, the traditional TPB model mainly emphasizes behavior decision-making processes based on rational cognition, while it has certain limitations in explaining habitual and context-dependent daily behaviors. First, CH reflect individuals’ automatic behavioral tendencies formed over long-term behavioral processes, capturing the “non-rational” and habitual components of unsafe cycling behavior, thereby complementing TPB’s focus on rational decision-making pathways. This is particularly relevant for Chinese college students, a group characterized by high-frequency cycling, where habitual behavioral patterns are more pronounced. Therefore, the inclusion of CH has clear theoretical and contextual significance. Second, risk perception (RP), defined as individuals’ subjective evaluation of potential risks, is a key cognitive variable in traffic behavior research. A substantial body of literature (7, 18, 19) has demonstrated a stable association between risk perception and unsafe traffic behaviors, indicating that RP is an important factor in explaining variations in risk-related behaviors. Incorporating RP into the model helps address the limitation of TPB in capturing the risk cognition dimension. Furthermore, the structural equation modeling results of this study show that CH (*β* = 0.15) and RP (*β* = −0.16) are both significantly associated with B, with effect sizes comparable to those of the core TPB variables. This finding suggests that these variables are not only statistically significant but also make substantive contributions to explaining unsafe cycling behavior, rather than merely improving model fit. In summary, the inclusion of cycling habits and risk perception not only extends TPB in terms of automatic behavior and risk cognition but also enables the model to better reflect the real-world context of cycling behavior among Chinese college students, thereby providing both theoretical and practical implications. This study innovatively considers both the indirect and direct relationships of cycling habits and risk perception with the crossing-campus cycling behavior of university students. This study is based on cross-sectional data, and the results only reflect statistical associations among variables, rather than allowing for causal inference. Although the selected university is somewhat representative in terms of its multi-campus structure, caution should be exercised when generalizing the findings to other universities, as traffic environments and cycling cultures may vary across regions.

As the cycling environment shifts from campus to campus outside, bad cycling habits from within campus can also transfer to campus outside areas (32, 33). As the potential risks increase, students with weaker RP are more likely to engage in unsafe cycling behaviors. Therefore, safety improvement measures for cross-campus cycling behavior of university students should focus on traffic safety education, promoting good cycling habits such as avoiding speeding, not running red lights, and wearing helmets. It is also crucial to enhance students’ ability to perceive risks on campus outside roads, thereby reducing the frequency of the cross-campus unsafe cycling behavior of university students.

## 6 Conclusion

### 6.1 Summary

This study innovatively considers both the indirect and direct relationships of CH and RP with unsafe cycling behavior. A survey questionnaire was designed to suit the cross-campus cycling student group, incorporating CH and RP as key factors. Valid data from 492 cross-campus cycling students were collected, and the improved TPB model was validated using SEM. The study quantitatively analyzed the influencing mechanisms of various factors on cross-campus unsafe cycling behavior of university students. The results show that:

1. AB, SN, PBC, CH, and RP are significantly correlated with BI, with path coefficients of 0.148, 0.195, 0.152, 0.127, and −0.133, respectively. BI, PBC, CH, and RP are significantly correlated with B, with path coefficients of 0.306, 0.203, 0.143, and −0.140, respectively. The improved TPB model shows a significant association with unsafe cross-campus cycling behavior of of university students, with an *R*^2^ of 53% for B variance.
2. Moreover, a comparison with the traditional TPB model reveals that the improved TPB model outperforms the traditional model in both fit and explanatory power, with a 11% improvement in the explained variance of B. This validates the reasonableness and effectiveness of the improved TPB model. The findings are of great significance for developing targeted interventions for the cross-campus cycling student group, reducing unsafe cycling behaviors, and enhancing road traffic safety.

### 6.2 Outlook

The scale of this study has situational mixing, which is to examine the migration effect of habits. Future research can develop a scale of pure cross-campus situations. The content of the measurement scale in this study may introduce justification bias or social desirability bias, which constitutes a limitation.

The experiments and methods of this study cannot infer the causal relationship between the addition of new variables and behavior, only the correlation.

The existing research attempts to incorporate CH and RP into the traditional TPB model and verifies the improvement of the model’s explanatory power through the structural equation model. In the future research, other potential variables such as road conditions, road infrastructure or emotional state may also improve the explanatory power of the model to a certain extent. Future research will explore the influence mechanism behind the deeper level. Although the modified model can effectively explain unsafe cross-campus cycling behavior of university students, due to the limitations of sample size and survey area, it is necessary to expand the survey area and increase the sample size in the future to further verify the reliability and explanatory power of the improved model. It should be noted that the sample of this study was obtained from a single university. Although the sample reflects, to some extent, the common characteristics of college students in multi-campus settings, differences in campus spatial structure, traffic organization, and cycling culture across universities may have an impact on the findings. Therefore, caution is warranted when generalizing the conclusions of this study to other contexts. Future research could employ multi-regional samples or conduct cross-campus comparative studies to further examine the robustness and external validity of the findings. Introduce methods to control for common method bias (CMB) in future analyses, such as the use of control variables, to reduce the impact of CMB on research results. Although the results of Harman’s test suggest that common method bias is not severe, its potential influence cannot be completely ruled out. Apply methods such as multiple regression analysis to explore the regression coefficients and mechanisms among variables in greater depth, thereby further validating the explanatory power of the model.

## Data Availability

The raw data supporting the conclusions of this article will be made available by the authors, without undue reservation.
